# Accurate SARC-F Score in Patients with Liver Disease

**DOI:** 10.3390/diagnostics13111959

**Published:** 2023-06-04

**Authors:** Masahiro Matsui, Akira Asai, Kosuke Ushiro, Saori Onishi, Tomohiro Nishikawa, Keisuke Yokohama, Hideko Ohama, Yusuke Tsuchimoto, Soo Ki Kim, Hiroki Nishikawa

**Affiliations:** 1 The Second Department of Internal Medicine, Osaka Medical and Pharmaceutical University, Takatsuki 569-8686, Osaka, Japan; akira.asai@ompu.ac.jp (A.A.); ushiro.1989@icloud.com (K.U.); kawakaminmin19910829@yahoo.co.jp (S.O.); tomohironisikawa5795@yahoo.co.jp (T.N.); keisuke.yokohama@ompu.ac.jp (K.Y.); hideko.ohama@ompu.ac.jp (H.O.); shibaraku6960@yahoo.co.jp (Y.T.); nishikawa_6392_0207@yahoo.co.jp (H.N.); 2 Liver Center, Osaka Medical and Pharmaceutical University, Takatsuki 569-8686, Osaka, Japan; 3 Department of Gastroenterology, Kobe Asahi Hospital, Kobe 653-0801, Hyogo, Japan; kinggold@kobe-asahi-hp.com

**Keywords:** SARC-F, liver disease, sarcopenia, predictor, discriminative ability

## Abstract

SARC-F is a well-accepted screening tool for sarcopenia. A SARC-F value of 1 point is reported to be more discriminating in identifying sarcopenia than 4 points (recommended cutoff point). The prognostic impact of the SARC-F score was investigated in patients with liver disease (LD, *n* = 269, median age = 71 years, 96 hepatocellular carcinoma (HCC) cases). Factors associated with SARC-F ≥ 4 points and SARC-F ≥ 1 point were also examined. In the multivariate analysis, age (*p* = 0.048), and Geriatric Nutritional Risk Index (GNRI) score (*p* = 0.0365) were significant factors linked to SARC-F ≥ 1 point. In our patients with LD, the SARC-F score is well correlated with the GNRI score. The 1-year cumulative overall survival ratio in patients with SARC-F ≥ 1 (*n* = 159) and SARC-F 0 (*n* = 110) was 78.3% and 90.1% (*p* = 0.0181). After excluding 96 HCC cases, similar tendencies were found (*p* = 0.0289). In the receiver operating curve (ROC) analysis based on the prognosis for the SARC-F score, the area under the ROC was 0.60. The sensitivity was 0.57, the specificity was 0.62, and the optimal cutoff point of the SARC-F score was 1. In conclusion, sarcopenia in LDs can be affected by nutritional conditions. A SARC-F score of ≥1 is more useful than a score of 4 in predicting the prognosis of patients with LD.

## 1. Introduction

Sarcopenia is a clinical condition characterized by loss of muscle mass and muscle function and is associated with frailty, cachexia, severe infections, and eventually death [1,2]. Age-related loss of muscle mass and strength is defined as primary sarcopenia, while disease-related loss of muscle mass and strength due to inadequate intake of protein, energy, etc. are classified as secondary sarcopenia [3,4]. Nutrient metabolism is often altered in patients with gastrointestinal diseases, and nutrient requirements and dietary intake differ from those of healthy individuals [5,6,7,8,9]. In other words, gastrointestinal diseases are considered representative of secondary sarcopenia [10,11]. Worldwide, the prevalence of sarcopenia in patients with liver disease (LD) is 5–13% in those in their 60s and 11–50% in those over 80 years of age, making it a frequent condition [12,13,14]. Sarcopenia in patients with LD can be associated with worse patient quality of life (QOL), a poorer prognosis, and higher healthcare costs [5,15,16,17]. LDs are representative diseases of secondary sarcopenia caused by the disease burden itself due to protein-energy malnutrition or other metabolic disorders [10]. Sarcopenia in LDs has been on the rise in recent years and can be a major concern in clinical practice.

The SARC-F is a five-question questionnaire and a globally recognized screening tool for sarcopenia [18,19]. Patients are asked to answer on a scale of 0 to 2 from “not at all difficult” to “very difficult” for muscle strength (S; weakness), walking aid (A; with or without a walking aid), rising from a chair (R), climbing stairs (C), and falling (F), and the total score (10-point scale) is calculated (recommended cutoff SARC-F score = 4 points). To diagnose sarcopenia in the elderly, the cutoff point of SARC-F 4 points shows low sensitivity (14–21%) and high specificity (90–94%), which is problematic as a screening method [19]. The reason for this (i.e., low sensitivity) may be the limited ability to extract subjects in the early stages of sarcopenia because the questions on the SARC-F focus primarily on symptoms related to functional decline associated with the progression of sarcopenia [20]. Attempts to improve the sensitivity of SARC-F to screen patients for sarcopenia have also been reported [21,22]. While a recent study reported that a SARC-F value of 1 point in patients with LD showed a greater discriminative ability to identify individuals at risk of sarcopenia than 4 points [23], as far as we are aware, little has been reported on the association between SARC-F scores of 1 or higher and LD patients. This clinical question seems worth clarifying the answer to. That is why we have come to this analysis.

## 2. Patients and Methods

### 2.1. Patients

We are one of the leading high-volume centers for LD in the country. Data on liver patients are continuously accumulated in our database. All hospitalized patients were required to complete the SARC-F questionnaire as a rule upon admission to the hospital, except for those who were unable to complete the questionnaire for reasons such as dementia. Between May 2020 and October 2022, 269 LD patients with SARC-F scores who were admitted to Osaka Medical and Pharmaceutical University can be found in our database. Baseline variables included were: age (years), gender, body mass index (BMI, kg/m^2^), hemoglobin (g/dL), C reactive protein (CRP, mg/dL), platelet count (×10^4^/mm^3^), serum albumin level (g/dL), serum bilirubin level (mg/dL), total lymphocyte count (/μL), and estimated glomerular filtration rate (eGFR, mL/min/1.73 m^2^). All personal information was kept anonymous and carefully protected.

### 2.2. SARC-F Score and Our Analysis

As mentioned earlier, SARC-F in each patient was calculated. SARC-F score and the percentage of patients with SARC-F ≥ 4 points or SARC-F ≥ 1 point were compared according to ALBI grade [24]. Next, the SARC-F score and the percentage of patients with SARC-F ≥ 4 points or SARC-F ≥ 1 point were compared according to the Geriatric Nutritional Risk Index (GNRI) grade (No risk, Mild risk, Moderate risk, and Severe risk [25]). The GNRI is a nutritional risk index that allows classifying patients according to the risk of morbidity and mortality in relation to pathologies in elderly patients that are often associated with malnutrition, as proposed by Bouillanne and colleagues [25]. It is a modified version (for the elderly) of the Nutritional Risk Index, a measure traditionally used to predict the risk of postoperative complications related to nutritional disorders [26,27].

The GNRI evaluates nutritional status using the ideal weight, actual weight, and serum albumin level [25,26,27]. GNRI is calculated by the following formula: GNRI = 1.487 × serum albumin (g/L) + 41.7 × body weight/ideal body weight (kg) [26,27]. GNRI < 82 is defined as severe nutritional risk, 82 < GNRI < 92 as moderate risk, 92 < GNRI < 98 as low risk, and GNRI > 98 as no risk [27,28], and is considered a useful prognostic factor in the elderly, patients with heart failure, dialysis, and malignancies [25,29,30]. Next, factors associated with SARC-F ≥ 4 points or SARC-F ≥ 1 point were examined using univariate and multivariate analyses. Next, a receiver operating characteristic (ROC) analysis of independent factors in the multivariate analysis for SARC-F ≥ 4 points or SARC-F score ≥ 1 point was performed. Next, the overall survival (OS) ratio was compared according to the SARC-F ≥ 4 points or the SARC-F ≥ 1 point. Finally, a ROC curve analysis for the SARC-F based on the prognosis was performed. Ethics approval was obtained from the Ethics Committee of Osaka Medical and Pharmaceutical University Hospital (approval number: 2021-109, approval date: 25 November 2021). The research is shown in detail on our website. Due to the retrospective nature of this study, informed consent from the patient was waived by the ethics committee of our hospital. Alternatively, an opt-out method was adopted for the current study. The protocol of this study strictly adhered to all provisions of the 1975 Declaration of Helsinki.

### 2.3. Statistical Considerations

For continuous parameters, the Student’s *t*-test or Mann–Whitney U test was applied as appropriate after confirming normality for two-group comparisons, and the ANOVA or Kruskal–Wallis test was applied as appropriate after confirming normality for multi-group comparisons. For categorical parameters, the Pearson χ^2^ test was applied to estimate group differences. For comparison of survival, the Kaplan–Meier method was applied and tested using the log-rank method. Data for continuous parameters were presented as median values (interquartile range, IQR). Multivariate logistic regression analysis linked to SARC-F ≥ 4 points or SARC-F ≥ 1 point was also performed to identify independent factors. The significance level was 0.05 by using JMP ver. 15 (SAS Institute Inc., Cary, NC, USA).

## 3. Results

### 3.1. Patient Baseline Characteristics

**Figure 1:**
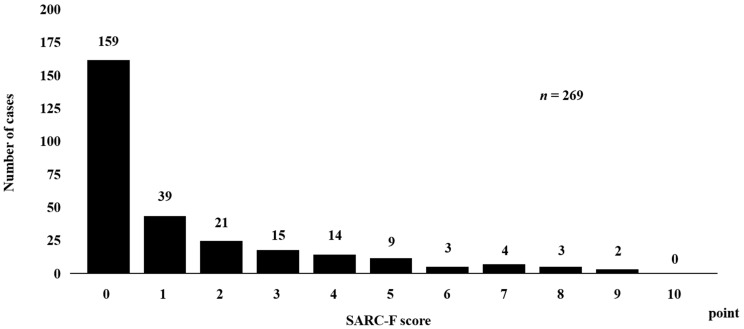
Number of patients according to SARC-F score.

Baseline characteristics for all cases (*n* = 269, 161 males and 108 females, median (IQR) age = 71 (64–78) years) are presented in Table 1. Hepatocellular carcinoma (HCC) was found in 96 cases (35.7%). The median (IQR) BMI was 23.1 (20.8–25.8) kg/m^2^. The median (IQR) serum alanine aminotransferase (ALT) level was 30.0 (18.0–55.0) IU/L. The median (IQR) serum albumin level was 3.8 (3.3–4.1) g/dL. The median (IQR) serum bilirubin level was 0.8 (0.5–1.2) mg/dL. The median (IQR) serum CRP level was 0.18 (0.06–0.64) mg/dL. The median (IQR) serum eGFR level was 65.0 (52.0–80.3) mL/min/1.73 m^2^. The median (IQR) serum total lymphocyte count level was 1244 (889–1649)/μL. The median (IQR) serum platelet count level was 14.9 (10.6–21.4) × 10^4^/mm^3^. The median (IQR) serum GNRI score was 98.9 (90.9–106.1). No risk of GNRI grade was found in 143 patients; a mild risk of GNRI grade was found in 52; a moderate risk of GNRI grade was found in 55; and a severe risk of GNRI grade was found in 19. The median (IQR) serum ALBI score was −2.38 (−2.03–−2.47). ALBI grade 1 was found in 104 patients, ALBI grade 2 in 152 patients, and ALBI grade 3 in 13 patients. The number of cases according to the SARC-F score is shown in Figure 1. SARC-F 0 points were observed in 159 cases (59.0%), and SARC-F ≥ 1 point in 110 cases (41.0%). SARC-F of < 4 points were observed in 234 cases (87.0%), and SARC-F of ≥ 4 points were observed in 35 cases (13.0%).

### 3.2. SARC-F Score According to ALBI Grade

**Figure 2:**
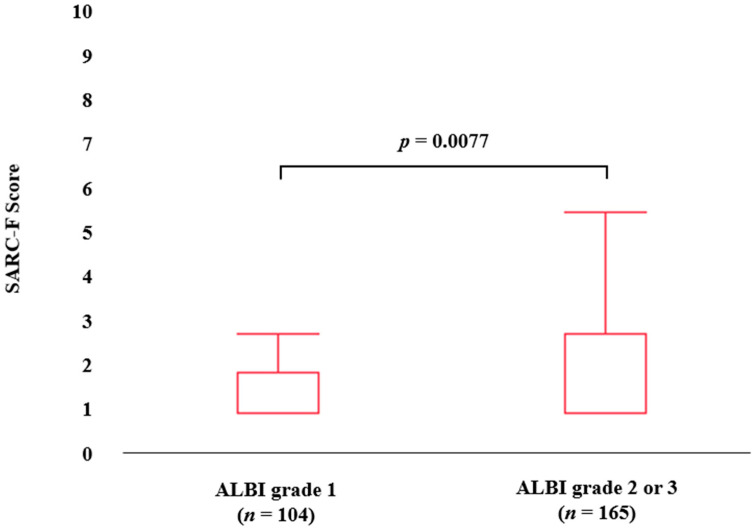
SARC-F score according to ALBI grade.

The median (IQR) SARC-F scores in groups of ALBI grade 1 (*n* = 104) and ALBI grade 2 or 3 (*n* = 165) were: 0 (0–1) in ALBI grade 1 and 0 (0–2) in ALBI grade 2 or 3, respectively (*p* = 0.0077) (Figure 2).

### 3.3. Percentage of Patients with SARC-F ≥ 4 Points or SARC-F ≥ 1 Point According to ALBI Grade

**Figure 3:**
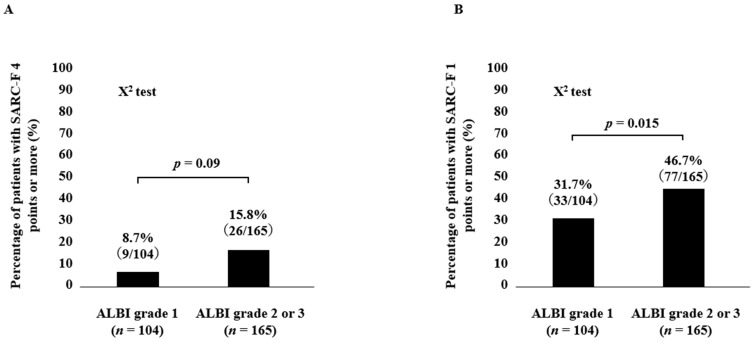
(**A**) Percentage of patients with a SARC-F score ≥ 4 points according to ALBI grade. (**B**) Percentage of patients with a SARC-F score ≥ 1 point according to the ALBI grade.

The percentage of SARC-F ≥ 4 points in groups of ALBI grade 1 and ALBI grade 2 or 3 was 8.7% (9/104) in ALBI grade 1 and 15.8% (26/165) in ALBI grade 2 or 3, respectively (*p* = 0.09) (Figure 3A). Likewise, the percentage of SARC-F ≥ 1 point in groups of ALBI grade 1 and ALBI grade 2 or 3 was 31.7% (33/104) in ALBI grade 1 and 46.7% (77/165) in ALBI grade 2 or 3, respectively (*p* = 0.015) (Figure 3B).

### 3.4. SARC-F Score According to GNRI Grade (No Risk, Mild Risk, Moderate Risk, and Severe Risk)

**Figure 4:**
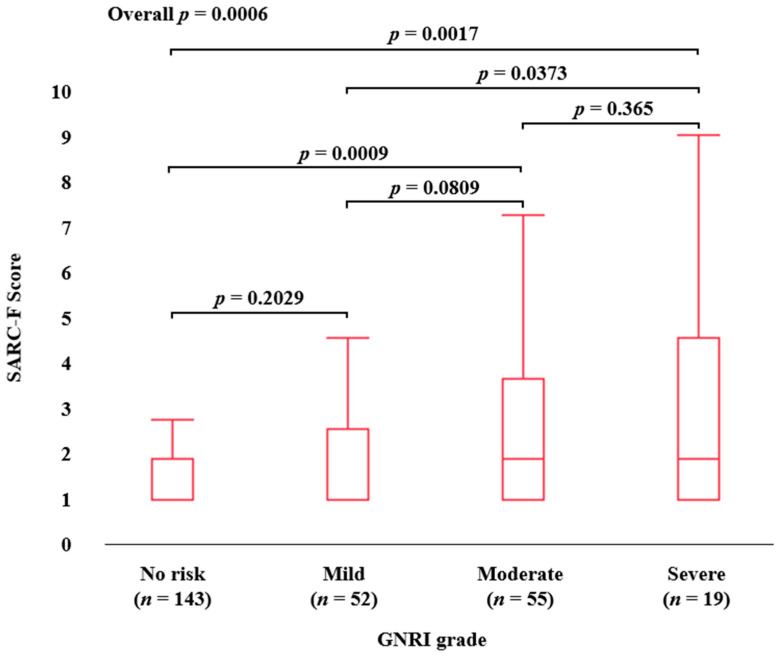
SARC-F score according to GNRI grade.

The median (IQR) SARC-F scores in groups of No risk, Mild risk, Moderate risk, and Severe risk patients as assessed by the GNRI grading system were: 0 (0–1) in No risk (*n* = 143), 0 (0–1.75) in Mild risk (*n* = 52), 1 (0–3) in Moderate risk (*n* = 55), and 1 (0–4) in Severe risk (*n* = 19), respectively (*p* values: No risk vs. Mild risk, *p* = 0.2029; No risk vs. Moderate risk, *p* = 0.0009; No risk vs. Severe risk, *p* = 0.0017; Mild risk vs. Moderate risk, *p* = 0.0809; Mild risk vs. Severe risk, *p* = 0.0373; Moderate risk vs. Severe risk, *p* = 0.365; overall *p* = 0.0006) (Figure 4).

### 3.5. Percentage of Patients with SARC-F ≥ 4 Points or SARC-F ≥ 1 Point According to GNRI Grade (No Risk, Mild Risk, Moderate Risk, and Severe Risk)

**Figure 5:**
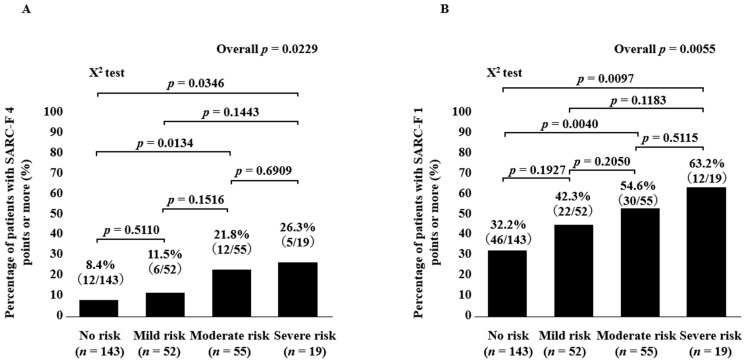
(**A**) Percentage of patients with SARC-F score ≥ 4 points according to GNRI grade. (**B**) Percentage of patients with SARC-F score > 1 point according to GNRI grade.

The percentage of SARC-F ≥ 4 points in groups of No risk, Mild risk, Moderate risk, and Severe risk patients was 8.4% (12/143) in No risk, 11.5% (6/52) in Mild risk, 21.8% (12/55) in Moderate risk, and 26.3% (5/19) in Severe risk, respectively (*p* values: No risk vs. Mild risk, *p* = 0.5110; No risk vs. Moderate risk, *p* = 0.0134; No risk vs. Severe risk, *p* = 0.0346; Mild risk vs. Moderate risk, *p* = 0.1516; Mild risk vs. Severe risk, *p* = 0.1443; Moderate risk vs. Severe risk, *p* = 0.6909; overall *p* = 0.0229) (Figure 5A). Likewise, the percentage of SARC-F ≥ 1 point in groups of No risk, Mild risk, Moderate risk, and Severe risk patients was 32.2% (46/143) in No risk, 42.3% (22/52) in Mild risk, 54.6% (30/55) in Moderate risk, and 63.2% (12/19) in Severe risk, respectively (*p* values: No risk vs. Mild risk, *p* = 0.1927; No risk vs. Moderate risk, *p* = 0.0040; No risk vs. Severe risk, *p* = 0.0097; Mild risk vs. Moderate risk, *p* = 0.2050; Mild risk vs. Severe risk, *p* = 0.1183; Moderate risk vs. Severe risk, *p* = 0.5115; overall *p* = 0.0055) (Figure 5B).

### 3.6. Univariate and Multivariate Analyses of Factors Associated with SARC-F ≥ 4 Points or SARC-F ≥ 1 Point

In the univariate analysis, age (*p* = 0.0002), eGFR (*p* = 0.0393), and GNRI score (*p* = 0.0010) were significant factors related to SARC-F ≥ 4 points (Table 2). The GNRI score (*p* = 0.0236) was the only independent factor related to SARC-F ≥ 4 points in the multivariate logistic regression analysis (Table 3). Hazard ratios (HRs) and 95% confidence intervals (CIs) for each factor are shown in Table 3. Likewise, in the univariate analysis, age (*p* < 0.0001), eGFR (*p* = 0.0004), GNRI score (*p* = 0.0035), and ALBI score were significant factors related to SARC-F ≥ 1 point (Table 2). Age (*p* = 0.048) and GNRI score (*p* = 0.0365) were independent factors related to SARC-F ≥ 1 point in the multivariate logistic regression analysis (Table 3). HRs and 95% CIs for each factor are shown in Table 3.

### 3.7. ROC Analysis of Independent Parameters for the SARC-F Score ≥ 4 Points or SARC-F Score ≥ 1 Point

The ROC analysis of GNRI in the multivariate analysis for SARC-F score ≥ 4 points was performed. The area under the ROC (AUC), the sensitivity, the specificity, and the optimal reference value of the GNRI score are demonstrated in Table 4. The AUC of the GNRI score for the SARC-F score ≥ 4 points was 0.67. Likewise, ROC analysis of independent factors in the multivariate analysis for SARC-F scores ≥ 1 point was conducted. The corresponding AUC, sensitivity, specificity, and best reference point for each factor are demonstrated in Table 4. Age involved the highest AUC for the SARC-F score ≥ 1 point (AUC = 0.66), followed by the GNRI score (AUC = 0.60).

### 3.8. The Cumulative OS Ratio According to SARC-F Score ≥ 4 Points or SARC-F Score ≥ 1 Point

**Figure 6:**
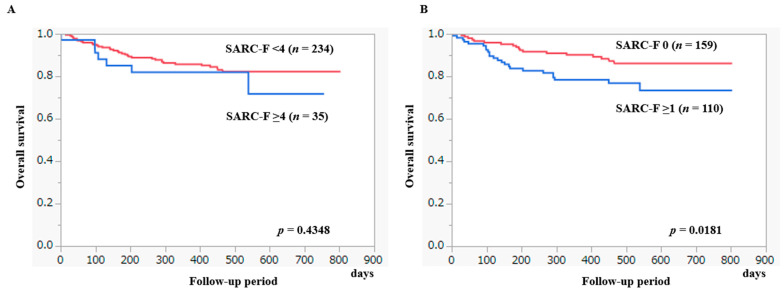
(**A**) The cumulative OS ratio according to the SARC-F ≥ 4 points. (**B**) The cumulative OS ratio according to the SARC-F ≥ 1 point.

Our median observation period was 448 days. In the observation period, 42 patients (16.0%) died. All deaths were liver-related. The 1-year cumulative OS ratio for all cases was 85.2%. The 1-year cumulative OS ratio in patients with SARC-F ≥ 4 (*n* = 35) and SARC-F < 4 (*n* = 234) was 82.0% and 85.7%, respectively (*p* = 0.4348, Figure 6A). Likewise, the 1-year cumulative OS ratio in patients with SARC-F ≥ 1 (*n* = 159) and SARC-F 0 (*n* = 110) was 78.3% and 90.1%, respectively (*p* = 0.0181, Figure 6B).

**Figure 7:**
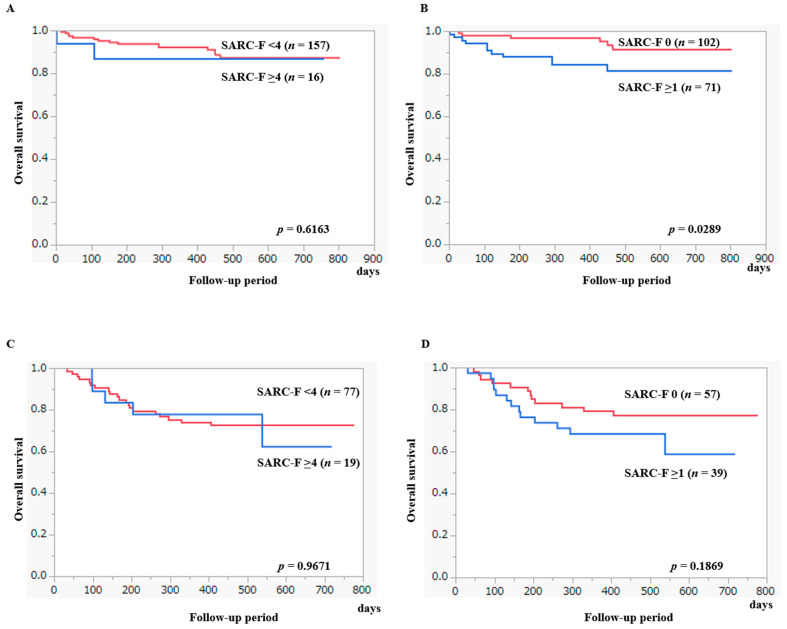
(**A**) The cumulative OS ratio according to the SARC-F ≥ 4 points after excluding HCC cases. (**B**) The cumulative OS ratio according to the SARC-F ≥ 1 point after excluding HCC cases. (**C**) The cumulative OS ratio according to the SARC-F ≥ 4 points in HCC cases. (**D**) The cumulative OS ratio according to the SARC-F ≥ 1 point in HCC cases.

After excluding 96 HCC cases, the 1-year cumulative OS ratio for all cases was 91.6%. The 1-year cumulative OS ratio in patients with SARC-F ≥ 4 (*n* = 16) and SARC-F < 4 (*n* = 157) was 87.1% and 92.1%, respectively (*p* = 0.6163, Figure 7A). The 1-year cumulative OS ratio in patients with SARC-F ≥ 1 (*n* = 71) and SARC-F 0 (*n* = 102) was 84.4% and 96.8%, respectively (*p* = 0.0289, Figure 7B).

In HCC cases, the 1-year cumulative OS ratio was 74.8%. The 1-year cumulative OS ratio in patients with SARC-F ≥ 4 (*n* = 19) and SARC-F < 4 (*n* = 77) was 77.8% and 74.0%, respectively (*p* = 0.9671, Figure 7C). The 1-year cumulative OS ratio in patients with SARC-F ≥ 1 (*n* = 39) and SARC-F 0 (*n* = 57) was 68.4% and 79.3%, respectively (*p* = 0.1869, Figure 7D).

### 3.9. ROC Analysis Based on the Prognosis for the SARC-F Score

**Figure 8:**
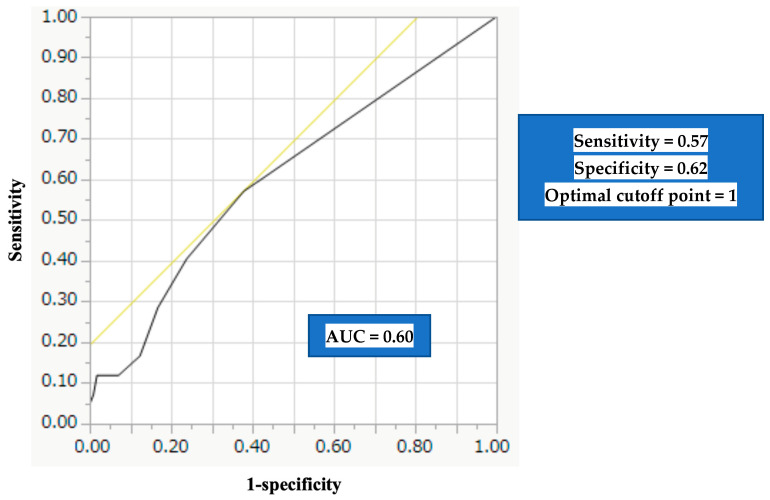
ROC analysis based on the prognosis for the SARC-F score.

In the ROC analysis based on the prognosis for the SARC-F score, the AUC was 0.60 (Figure 8). Corresponding sensitivity and specificity were 0.57 and 0.62, and the optimal cutoff point of the SARC-F score was 1 (Figure 8).

## 4. Discussion

More than a quarter century has passed since the disease concept of sarcopenia was first proposed, and subsequent research has led to remarkable academic developments in this field [31]. In the field of public health, sarcopenia has received a lot of attention recently because of its close association with clinical outcomes (i.e., falls, fractures, infections, frailty, survival) [1,2,15,17,32,33,34,35]. In order to diagnose sarcopenia, skeletal muscle mass must be assessed, but imaging tests such as computed tomography and bioelectrical impedance analysis for skeletal muscle mass assessment are often unavailable in smaller clinics. In that sense, SARC-F is convenient and useful, and its use as an initial screening is recommended in the current international guidelines [3,35,36,37]. Yang et al. reported a very good AUC of 0.86 and 0.90 for men and women, respectively, using the recommended reference point of SARC-F (i.e., 4 points) for the screening of sarcopenia [36]. Thus, SARC-F seems to be very helpful for sarcopenia screening, although the sensitivity of SARC-F for sarcopenia is unsatisfactory low [3,19,36,37]. Additionally, SARC-F has recently been reported to be well correlated with all-cause mortality and all-cause-specific mortality [38].

In recent years, a SARC-F score of 1 point in patients with LD has been shown to have a higher ability to identify sarcopenia than the conventionally used score of 4 points [23]. In our data, the percentage of ALBI grade 1 (104 cases) and ALBI grade 2 or 3 (165 cases) with SARC-F ≥ 4 points was 8.7% for ALBI grade 1 and 15.8% for ALBI grade 2 or 3, which was not significantly different (*p* = 0.09). While the percentage of patients with SARC-F ≥ 1 point in ALBI grade 1 and ALBI grade 2 or 3 was 31.7% and 46.7%, respectively (*p* = 0.015), in patients with LD, a SARC-F cutoff of 1 point may clarify the relationship between liver function and sarcopenia. In our results, the SARC-F score and the percentage of patients with a SARC-F score ≥ 4 or a SARC-F score ≥ 1 were well stratified by the nutritional marker as assessed by the GNRI score in all analyses. These findings suggest that the SARC-F score may reflect the nutritional status of LD patients and may provide a clue to the relationship between malnutrition and sarcopenia in patients with LD. GNRI score (*p* = 0.0236) was an independent factor related to SARC-F ≥ 4 points in the multivariate logistic regression analysis. Likewise, age (*p* = 0.048) and GNRI score (*p* = 0.0365) were independent factors related to SARC-F ≥ 1 point in the multivariate logistic regression analysis. Our results imply that the SARC-F ≥ 1 in LDs is closely associated with not only the primary factor (aging) but also a secondary factor such as the nutritional factor. We have previously reported that patients with advanced gastrointestinal cancers with SARC-F < 4 points have a better prognosis than patients with SARC-F ≥ 4 points [6]. While we found no difference in prognosis in patients with advanced HCC with SARC-F ≥ 4 vs. <4, a SARC-F cutoff score of 4 may not be a sufficiently reliable predictor of prognosis in patients with LD [6]. In terms of survival, the 1-year cumulative OS ratio for all patients in this study was 85.2%. The 1-year cumulative OS ratios for patients with SARC-F ≥ 4 and SARC-F < 4 was 82.0% and 85.7%, respectively, which were not significantly different (*p* = 0.4348). The 1-year cumulative OS ratio for patients with SARC-F ≥ 1 and SARC-F 0 were 78.3% and 90.1%, respectively, with statistical significance (*p* = 0.0181). After excluding HCC cases, similar trends were found. In the ROC analysis based on the prognosis for the SARC-F score, the AUC was 0.60, the corresponding sensitivity and specificity were 0.57 and 0.62, and the optimal reference point for the SARC-F score was 1. When the cutoff point of the SARC-F score was set at 4, the corresponding sensitivity and specificity were 0.17 and 0.88, denoting a significant loss of sensitivity. As mentioned above, a cutoff of 4 points on the SARC-F for sarcopenia in patients with LD has relatively low sensitivity, and a cutoff of 1 point may be useful. The recommended SARC-F ≥ 4 points may only identify patients with more advanced sarcopenia, while SARC-F ≥ 1 point may be ideal for the early detection of sarcopenia, especially in patients with LD. Considering these study results, the optimal reference point for the SARC-F score should be carefully considered. It is obvious that lowering the cutoff to 1 from 4 would reduce the number of missed cases of sarcopenia. Ideally, the cutoff point should be at a point where sensitivity and specificity are optimally balanced, but considering SARC-F’s position as a screening tool, better sensitivity may be better [39]. Alternatively, a modified version of SARC-F could be used to increase detection sensitivity for sarcopenia [40].

It must be stated that this study has several limitations. First, this is a single-center, cross-sectional Japanese study that, by its nature, is retrospective. We did not examine lifestyle habits in other ethnic groups other than the Japanese. In addition, the SARC-F is a self-report questionnaire that indicates the likelihood of risk of sarcopenia, and data on the number of patients with definite sarcopenia were not included in the present analysis. Finally, in terms of survival analysis, various treatments for background LDs have been given during the course of the disease, which may affect the prognosis, also creating biases. Despite these limitations, the results of this study indicate that a baseline SARC-F score of 1 correlates well with the GNRI score in patients with LD and may be a predictor of prognosis.

## 5. Conclusions

Sarcopenia in patients with LD can be affected by nutritional condition. A SARC-F ≥ 1 score is useful in predicting the prognosis of patients with LD. Clinicians should be aware of these.

## Figures and Tables

**Table 1:** Baseline characteristics (*n* = 269).

	Number or Median (IQR)
Age (years)	71.0 (64.0, 78.0)
Gender, male/female	161/108
BMI (kg/m^2^)	23.1 (20.8, 25.8)
ALT (IU/l)	30 (18.0, 55.0)
Serum albumin (g/dL)	3.8 (3.3, 4.1)
Total bilirubin (mg/dL)	0.8 (0.5, 1.2)
CRP (mg/dL)	0.18 (0.06, 0.64)
eGFR (mL/min/1.73 m^2^)	65 (52.0, 80.3)
Hb (g/dL)	12.8 (11.5, 14.1)
Total lymphocyte count (/μL)	1244 (889, 1649)
PT (%)	88 (76.0, 99.0)
Platelet count (×10^4^/mm^3^)	14.9 (10.6, 21.4)
SARC-F score, 0/1 or more	159/110
GNRI score	98.9 (90.9, 106.1)
GNRI grade, No risk/Mild risk/Moderate risk/Severe risk	143/52/55/19
ALBI score	−2.48 (−2.76, −2.03)
ALBI grade, 1/2/3	104/152/13

BMI; body mass index, ALT; alanine aminotransferase, CRP; C reactive protein, eGFR; estimated glomerular filtration rate, Hb; hemoglobin, PT; prothrombin time, GNRI; Geriatric Nutritional Risk Index, ALBI; albumin-bilirubin, IQR; interquartile range.

**Table 2:** Univariate analysis of factors linked to SARC-F ≥4 points or SARC-F ≥1 point.

	**SARC-F < 4 Points** **(*n* = 234)**	**SARC-F ≥ 4 Points** **(*n* = 35)**	***p* Value**
Age (years)	70.5 (62.0, 77.0)	78.0 (71.0, 80.0)	0.0002
Gender, male/female	140/94	21/14	0.9846
BMI (kg/m^2^)	23.3 (20.9, 25.8)	22.4 (19.0, 25.4)	0.0993
CRP (mg/dL)	0.18 (0.07, 0.65)	0.19 (0.05, 0.53)	0.8727
eGFR (mL/min/1.73 m^2^)	65.0 (53.0, 81.0)	58.0 (43.0, 73.0)	0.0393
Lymphocyte count (/μL)	1282.0 (892.5, 1683.0)	1107.5 (816.0, 1356.5)	0.0994
Platelet count (×10^4^/mm^3^)	152.0 (109.0, 219.3)	135.0 (91.0, 176.0)	0.0944
GNRI score	99.8 (92.7, 107.1)	93.2 (86.3, 100.9)	0.0010
ALBI score	−2.49 (−2.77, −2.07)	−2.36 (−2.60, −1.71)	0.0919
	**SARC-F 0 Point** **(*n* = 159)**	**SARC-F ≥ 1 Point** **(*n* = 110)**	***p* Value**
Age (years)	69.0 (59.0, 76.0)	75.0 (69.8, 80.0)	<0.0001
Gender, male/female	101/58	60/50	0.160
BMI (kg/m^2^)	23.2 (20.9, 25.3)	22.9 (20.7, 25.8)	0.9936
CRP (mg/dL)	0.15 (0.06, 0.44)	0.22 (0.07, 0.70)	0.117
eGFR (mL/min/1.73 m^2^)	69.0 (56.0, 84.0)	59.0 (46.8, 73.5)	0.0004
Lymphocyte count (/μL)	1300.5 (899.5, 1654.8)	1121.0 (810.5, 1629.0)	0.0870
Platelet count (×10^4^/mm^3^)	153.0 (110.0, 221.0)	144.5 (98.5, 201.0)	0.3091
GNRI score	100.5 (94.2, 106.6)	96.7 (88.8, 104.5)	0.0035
ALBI score	−2.52 (−2.82, −2.17)	−2.41 (−2.66, −1.84)	0.0232

Data are presented as number or median value (interquartile range). BMI; body mass index, CRP; C reactive protein, eGFR; estimated glomerular filtration rate, GNRI; Geriatric Nutritional Risk Index, ALBI; albumin-bilirubin.

**Table 3:** Multivariate analyses of factors associated with SARC-F ≥4 points or SARC-F ≥1 point.

**SARC-F Score ≥ 4 Points**	**Multivariate Analysis**		
	**OR**	**95% CI**	***p* Value**
Age (per one year)	0.9606	0.917–1.006	0.0883
Gender (female)	1.0089	0.447–2.275	0.9829
BMI (per one kg/m^2^)	0.7672	0.532–1.106	0.1560
CRP (per one mg/dL)	0.9505	0.807–1.120	0.5440
Lymphocyte count (per one/μL)	1.0001	0.999–1.001	0.7116
Platelet count (per one ×10^4^/mm^3^)	1.0049	0.999–1.011	0.1164
eGFR (per one mL/min/1.73 m^2^)	1.0024	0.984–1.021	0.7963
GNRI score (per one)	1.2144	1.026–1.437	0.0236
ALBI score (per one)	13.247	0.812–216.1	0.0697
**SARC-F Score ≥ 1 Point**	**Multivariate Analysis**		
	**OR**	**95% CI**	***p* Value**
Age (per one year)	0.975	0.951–1.000	0.0480
Gender (female)	1.626	0.944–2.802	0.0799
BMI (per one kg/m^2^)	0.8416	1.034–1.188	0.1003
CRP (per one mg/dL)	1.0482	0.130–8.442	0.9647
Lymphocyte count (per one/μL)	0.9999	0.999–1.000	0.8545
Platelet count (per one ×10^4^/mm^3^)	1.0001	0.997–1.004	0.7589
eGFR (per one mL/min/1.73 m^2^)	1.0078	0.995–1.021	0.2276
GNRI score (per one)	1.1085	1.006–1.221	0.0365
ALBI score (per one)	2.6632	0.589–12.03	0.2031

OR; odds ratio, CI; confidence interval, BMI; body mass index, ALT; alanine aminotransferase, CRP; C reactive protein, eGFR; estimated glomerular filtration rate, GNRI; Geriatric Nutritional Risk Index, ALBI; albumin-bilirubin.

**Table 4:** Receiver operating curve analysis of independent parameters for SARC-F score ≥ 4 points or SARC-F score ≥ 1 point.

**SARC-F Score ≥ 4 Points**	**AUC**	**Sensitivity (%)**	**Specificity (%)**	**Cutoff Point**
GNRI score	0.67	60.0	70.1	94.7
**SARC-F Score ≥ 1 Point**	**AUC**	**Sensitivity (%)**	**Specificity (%)**	**Cutoff Point**
Age	0.66	58.0	69.0	72.0
GNRI score	0.60	57.0	62.0	98.0

GNRI; Geriatric Nutritional Risk Index, AUC; area under the receiver operating characteristics curve.

## Data Availability

The data presented in this study are available on request from the corresponding author. The data are not publicly available due to the personal information.

## Abbreviations

LD; liver disease, QOL; quality of life, BMI; body mass index, CRP; C reactive protein, eGFR; esti-mated glomerular filtration rate, IQR; interquartile range, GNRI; geriatric nutritional risk index, ROC; receiver operating charac-teristic, OS; overall survival, HCC; hepatocellular carcinoma, HR; hazard ratio, CI; confidence interval, AUC; area under the ROC.
